# Supplementary material for: Association between osteoporosis and periodontal disease among menopausal women: The 2013–2015 Korea National Health and Nutrition Examination Survey
Source: PLoS One. 2022 Mar 17;17(3):e0265631. doi: 10.1371/journal.pone.0265631 (PMC8929583; doi:10.1371/journal.pone.0265631)
Supplement: Attachment — Submitted filename: response to reviewers.docx [file pone.0265631.s002.docx]

| Reviewer #1 | | | |
| --- | --- | --- | --- |
| Page | Line | *Comment* | Response |
|  |  | *English Language: In general, the text should be revised with the intention of making the reading more fluid. Although the manuscript is understandable, it would benefit a lot from careful editing by an expert in writing scientific English.* | Thank you for highlighting this issue. I agree with this and the manuscript has now been edited by a native English speaker who is an expert in writing scientific English. |
| 1 | 1 | *Title: Well written, makes the reader interested in the full article.  Keywords: I did not find the term “Menopausal transition” in Mesh. I suggest “Menopause”, instead. I also suggest inserting the term “Korea National Health and Nutrition Examination Survey” among the keywords.* | Agree. I have made the suggested changes. |
| 1  4 | 21-23  77-79 | *Methods: I suggest inserting the study design: “this cross-sectional study aimed...”This sentence, “formerly Korea Centers for Disease Control and Prevention”, is not necessary for the Abstract; just in the Method section. I suggest substitute it for the period of the KNHANES, eg: “...conducted by the Korea Disease Control and Prevention Agency (KDCA) from 2013 to 2015”.* | Thank you. I have made the changes you have suggested. |
| 2 | 40-42 | *It’s important to add information on the variable ‘duration after menopause’, categorized as 0-4, 5-9, and ≥ 10 years.*  *Please, also add information on statistic analysis.* | I agree with you and have made the required changes. |
| 2 | 39-42 | *2- Results: The authors state that “The osteoporosis group had an adjusted OR of 1.25 (95% CI:1.00-1.56) for developing periodontal disease”. The term “for developing” could be used if the study had a longitudinal design. With a cross-sectional design, authors should limit their statement to “for presenting periodontal disease”. The same for the next line of Results.* | I have made the required changes. |
| 2 | 39-42 | *Conclusions: The term “risk factor” in Epidemiology is frequently involved with causality and is better linked to cohort studies. I suggest that “risk factor”, in “Osteoporosis could be considered a risk factor of periodontal disease”, should be substituted by “Osteoporosis was an associated factor with periodontal disease”, or “Osteoporosis could be considered a risk indicator of periodontal disease in the studied population. Such effect was...”* | I have changed the text in light of your comments. |
| 2  3 | 43-  74 | *Introduction I recommend the authors restructure/re-elaborate their introduction. Unfortunately, the English language shows low quality. Again, I strongly recommend careful proofreading. Throughout the whole manuscript, the misuse, misspellings are noted.* | Thank you. The Introduction has been rewritten and the whole manuscript has been edited by a professional English language Editor. |
| 2 | 44 | *The first paragraph should introduce the main topic involved in the study: osteoporosis. So, the second paragraph could be the first one, followed by the others information.* | I agree with you. The Introduction has been rewritten as per your suggestion. |
| 2 | 44- | ** Not only age and gender are related to the decrease in BMD. Factors like smoking, diabetes, low body mass index, glucocorticoids use, rheumatoid arthritis, and others, are related to low BMD Following the sentence above: “The cause of this is related to a postmenopausal decrease in estrogen level forming bone resorbing cytokines, such as RANKL, TNF-α, and interleukin 1, which could contribute to the onset of a series of diseases, including osteopenia and osteoporosis”. Then, replace the first paragraph on following: " According to the 2020 census by Statistics Korea, the population size of Koreans aged 65 years or older is approximately 8.13 million, accounting for 15.7% of the total population. In particular, females account for 76.2% of the elderly population with 4.61 million. As a result of reaching such aging society, osteoporosis has garnered social and medical attention.”* | I have edited the manuscript to address your comments. |
| 3 | 63 | *“Osteoporosis and periodontal disease are multifactorial chronic diseases with systemic and localized bone loss”, add the term “respectively”, since there is no evidence that periodontal disease causes systemic bone loss.* | Thank you for highlighting this issue, which has been addressed in the revised manuscript. |
| 4 | 77 | *“this study used raw data from the sixth (2013-2015) Korea National Health and Nutrition Examination Survey (KNHANES-VI) conducted by the Korea Disease Control and Prevention Agency (KDCA)”: this belongs to Materials and Methods.* | I agree and have moved the text to the Materials and Methods section accordingly. |
| 4 | 77 | *1-Please, begin this section with the study design. “This was a cross-sectional study...”* | This change has now been made. |
| 5 | 105 | *About the periodontal exam: Please, describe how many examiners were involved in the data collection. Were they calibrated? Is there a measure of reliability, such as the kappa coefficient? Where the exams were performed (at dental offices?) Please, provide more details on CPITN: all present teeth were examined and the higher observed CPITN score was attributed to the sextant, or only the index teeth were examined and the higher observed CPITN score attributed to the sextant?* | This detail has been elaborated on in the revised manuscript. |
| 4 | 96 | *The assessment of bone mass needs to be clarified. Was the densitometry performed using Dual-energy X-ray absorptiometry (DXA)? The authors do not define the T-score used to classify osteoporosis. Was it ≤-2,5? Which bone sites were considered: lumbar spine, femoral neck, and total fêmur?* | Thank you for your comments. The manuscript has been revised to address these questions. |
| 5 | 121 | *Statistical analysis: Please, clarify lines 94 to 97.*  *Information on the statistical tests used is lacking. Example, in table 2: Kruskal-Wallis, Mann Whitney, Qui-Square?* | The statistical tests are now elucidated on in the revised manuscript. |
| 9 | 170 | *Association between osteoporosis and periodontal disease Please, the authors should express, in the text, what is the meaning of the OR below 1.0, such as: .79 (95% CI:.64-.99), .76 (95% CI:.62-.94), .63 (95% CI:.42-.94) and .94 (95% CI:.76-1.16) for developing periodontal disease. Would these factors be protective factors? Which percentage?* | The text has been revised to clarify the results. |
| 6  8  10 | 150  166  186 | *please, the titles could be more elaborated.* | The titles have been changed accordingly. |
| 11 | 199 | *Discussion: Line 157: Please, I recommend start the Discussion in a different manner, eg: Based on the analysis of naturally menopausal women aged 45-60 years in this study, .... Line 163: [14] was not a prospective study. Line 165 [12]; please, the investigation of this report was performed in another way. Check the results and conclusion and re-elaborate the sentence. Line 185: decreased or increased? Line 200 and others: the authors need to write the name of the authors included in the manuscript in a standard way.* | Thank you for this comment. I have revised the Discussion section accordingly. |
| 12 | 218-232 | *The higher the age, the higher the systemic bone loss. Why would the duration 0-4 years be associated with periodontal disease, and the higher periods not? This is the main topic that should be involved in this section. Please, discuss this important finding.* | Thank you for this insightful comment. To answer your question, in individuals who are 3–4 years after menopause, it is hypothesized that the increased risk of periodontal disease may be due to a decrease in bone density due to a sudden lack of estrogen. This has been mentioned as a possible causative factor in the manuscript. |
| 13 | 247 | *Line 205: “Drinking and smoking are key causes of periodontal disease” Please, the authors should not state that drinking is a key factor because it is not evidenced as a risk factor for periodontal disease. I recommend they should be more careful and, at the maximum, state that drinking may be an associated factor related to periodontal disease. Another way is “Drinking and smoking were considered causes for periodontal disease according to some authors...” Drinking has shown (instead of ‘has’) a negative effect on maintaining the balance...”* | I agree with you and have modified the manuscript text accordingly. |
| 13 | 250 | *Line 209: Aggregatibacter. Please, correct.* | This has been modified as requested. |
| 13 | 258 | *Limitations: 1- The cut-off for osteoporosis was based on a T-score (the authors should provide information on this in Materials and Methods). This means that the intermediate scores, even those representing advanced osteopenia, close to osteoporosis, were included in the normal BMD group. Considering osteopenia, even severe cases, as a normal group, is a limitation of the study. Explore limitations of the CPITN.* | The limitations of the study have been expanded on in the revised manuscript, as per your suggestion. |
| 1 | 15 | *the abstract should mention that the models controlled for confounding variables.* | This has been addressed in the revised abstract. |
|  |  | *I encourage the author to run an unweighted analysis and compare it to the weighted analysis. If they are very close, I would recommend just presenting the unweighted analysis. It is unclear if the efforts to weight an analysis of such a small subgroup is a good idea or not.* | Thank you for pointing this out. I agree with this comment. Comparing the unweighted analysis to the weighted equation, the results were very similar. |
| 7 | 165 | *it would be easier to interpret if Tables 1 and 2 were combined (author could include an “all” column in Table 2 to combine both tables).* | Table 1 and 2 seemed to overlap, so Table 1 was deleted and Table 2 was modified. |
| 10 | 194 | *the style of presentation of the model (where just the exposure variables of interest have their ORs reported) is ideal in Table 4, and my suggestion would be to combine tables 3 and 4 and only present the ORs for the exposure variables (and not interpret them – just for the sake of brevity).* | In Table 4, a binary logistic regression analysis was performed after stratifying the ‘postmenopausal period (years)’ variable to understand the relationship between osteoporosis and periodontal disease according to the postmenopausal period, separate from Table 3. |
| 11 | 198 | *The discussion is very good in that it interprets the results against the existing literature, but there are some awkward sentences. I will recommend minor corrections and then when I see the next version, I will give more careful feedback about the discussion.* | Thank you for your comment. I have revised the discussion, which has been edited by a professional English language Editor. I hope that the revised discussion is acceptable. |
