# Supplementary material for: Association between osteoporosis and periodontal disease among menopausal women: The 2013–2015 Korea National Health and Nutrition Examination Survey
Source: PLoS One. 2022 Mar 17;17(3):e0265631. doi: 10.1371/journal.pone.0265631 (PMC8929583; doi:10.1371/journal.pone.0265631)
Supplement: Attachment — Submitted filename: renamed_a2624.docx [file pone.0265631.s003.docx]

| Reviewer #1 | | | |
| --- | --- | --- | --- |
| Page | Line | *Comment* | Response |
| 1 | 22 | *Methods: I suggest inserting the period of the KNHANES, “Korea National Health and Nutrition Examination Survey, from 2013 to 2015”, the final population...* | I agree with you and have made the required changes. |
| 2 | 34 | *Conclusions: I suggest using past tense instead of the present tense: “Osteoporosis was an associated factor with periodontal disease, and the association...”* | I agree with you and have made the required changes. |
| 3 | 60 | *Line 57:“According to existing studies, systemic BMD loss due to osteoporosis can also affect alveolar bone density and periodontal disease. In a study on postmenopausal women...” Line 60 “In the same context, it has been reported that there is an association between osteoporosis and periodontal disease among postmenopausal women ...”*  *These sentences bring the same information. It seems to me that the authors wanted to talk about the bidirectional via between osteoporosis and periodontal disease. If so, in line 60/61, it has to become clear, maybe inserting the term “inverse”, eg “it has been reported that there is an inverse association between osteoporosis and periodontal disease...”* | I agree with you and have made the required changes. |
| 6  8  10 | Table1  Table2  Table3 | *Results: Please, correct terms in footnotes, like “alchol” and “consumpion”. Besides, some parenthesis is missing in the table* | I agree with you and have made the required changes. |
| 12  13 | 243  249 | *Discussion:*  *Line 239: Please, correct “CPI”* | Thank you. I have made the changes you have suggested. |
